# Protein 3D Hydration: A Case of Bovine Pancreatic Trypsin Inhibitor

**DOI:** 10.3390/ijms232314785

**Published:** 2022-11-26

**Authors:** Sergey E. Kruchinin, Ekaterina E. Kislinskaya, Gennady N. Chuev, Marina V. Fedotova

**Affiliations:** 1 G.A. Krestov Institute of Solution Chemistry, The Russian Academy of Sciences, Akademicheskaya St., 1, 153045 Ivanovo, Russia; 2 Department of Fundamental and Applied Chemistry, Institute of Mathematics, Information Technology and Science, Ivanovo State University, Ermak St., 39, 153025 Ivanovo, Russia; 3 Institute of Theoretical and Experimental Biophysics, The Russian Academy of Sciences, Institutskaya St., Pushchino, 142290 Moscow, Russia

**Keywords:** protein, bovine pancreatic trypsin inhibitor, hydration structure, molecular dynamics, 3D-RISM integral equation method

## Abstract

Characterization of the hydrated state of a protein is crucial for understanding its structural stability and function. In the present study, we have investigated the 3D hydration structure of the protein BPTI (bovine pancreatic trypsin inhibitor) by molecular dynamics (MD) and the integral equation method in the three-dimensional reference interaction site model (3D-RISM) approach. Both methods have found a well-defined hydration layer around the protein and revealed the localization of BPTI buried water molecules corresponding to the X-ray crystallography data. Moreover, under 3D-RISM calculations, the obtained positions of waters bound firmly to the BPTI sites are in reasonable agreement with the experimental results mentioned above for the BPTI crystal form. The analysis of the 3D hydration structure (thickness of hydration shell and hydration numbers) was performed for the entire protein and its polar and non-polar parts using various cut-off distances taken from the literature as well as by a straightforward procedure proposed here for determining the thickness of the hydration layer. Using the thickness of the hydration shell from this procedure allows for calculating the total hydration number of biomolecules properly under both methods. Following this approach, we have obtained the thickness of the BPTI hydration layer of 3.6 Å with 369 water molecules in the case of MD simulation and 3.9 Å with 333 water molecules in the case of the 3D-RISM approach. The above procedure was also applied for a more detailed description of the BPTI hydration structure near the polar charged and uncharged radicals as well as non-polar radicals. The results presented for the BPTI as an example bring new knowledge to the understanding of protein hydration.

## 1. Introduction

The water–protein interactions are crucial for the structure, dynamics, folding, and functionality of proteins. A fundamental understanding of these interactions is a necessary and important step toward characterizing the role of water in solvated biological systems. Many experimental studies have been performed on protein hydration (see, for instance, [1,2,3,4,5,6,7,8]). Despite these advances, protein–water interactions are still not fully understood because of the considerable difficulty associated with their experimental study. In particular, the very complex surfaces of biological macromolecules and their hydration environment are the most poorly defined parts of structures obtained by crystallography or NMR because they are often disordered or dynamically averaged [4]. Likewise, X-ray and N-diffraction measurements, even with modern equipment, face the problem of limited resolution. Not all the water molecules at the protein surface are strongly bound to specific protein sites; some are exchanged with the bulk solvent. As a result, one can determine a location only for some of the nearest waters with significant residence time close to some specific protein positions [9]. A way to overcome experimental difficulties and give a detailed description of biomolecule solvation is to use modern molecular dynamics (MD) simulations [10,11,12,13]. Another attractive option can be found in the three-dimensional reference interaction site model (3D-RISM)—an integral equation method of the statistical theory of liquids [14,15,16,17,18,19,20,21,22,23,24,25]. Both methods are known to be powerful techniques providing a rather accurate 3D-molecular picture of protein hydration at a level of detail far beyond experimental reach [9,10,12,26,27,28]. MD simulations provide a high-resolution view of the motions of biomolecules in the form of continuous trajectories and from it a description of various properties including solvation at atomic or molecular levels. The 3D-RISM theory takes a statistical mechanics route describing the solvation effects on the molecular-atom level in terms of spatial distribution functions (SDFs), g(r), for solvent sites (atoms) around the reference entire solute molecule. In addition to application to the study of solvation, the proposed methods are widely used in protein science, for example, to investigate protein–protein interactions and molecular recognition [9,29,30,31,32,33], protein–ligand binding [15,21,27,34], or the role of bridging water molecules within a protein complex [28].

In this contribution, we present the results of a 3D-hydration study for protein bovine pancreatic trypsin inhibitor (BPTI) as an example. To this end, we have used 2-microsecond (2-μs) all-atom MD simulations and 3D-RISM calculations which are mutually complementary to each other. BPTI is a widely investigated protein with a well-established crystal structure defined from X-ray, N-diffraction, and NMR measurements [35,36,37,38]. BPTI has been found in a variety of organs and tissues in mammals including in bovine (see, for instance, [39,40,41]) and also in other vertebrates and in invertebrates (see, for instance, [42,43,44,45,46]). The Kunitz-type inhibitor, BPTI, is a member of the serine protease family of inhibitors [47]. BPTI has a relatively broad specificity in that it can inhibit several kinds of digestive enzymes, such as trypsin, chymotrypsin, plasmin and kallikrein [48]. BPTI’s function is the suppression of protein digestion, i.e., the breakdown of proteins into their peptide building blocks, by means of inhibiting the action of the enzyme, in particular, trypsin, which is produced in the bovine pancreas. Under its function, BPTI forms stable complexes with the enzymes that it inhibits. In particular, as found [49,50], BPTI binds to trypsin with high affinity. In the trypsin–BPTI complex, BPTI prevents the water molecules from penetrating the active site [50]. Complex formation is accompanied by the desolvation of binding partners. It was found [29] that the desolvation of hydrophobic patches promotes the binding, whereas the desolvation of charged and polar residues slows it down As a result, an exclusion of water from the contact interface under protein–protein association increases the role of van der Waals interactions [51].

BPTI is a small, compactly folded, globular protein of 58 amino-acid residues with a mass of 6512 Da stabilized by three disulfide bonds [47,52,53]. The latter provides the formation of a compact and very stable tertiary structure. Even in an aqueous solution, the BPTI structure is close to the crystal one (see, for instance, [37]). The small size, high stability, and solubility of BPTI make it an attractive model system featured in many experimental and theoretical studies. Another aspect, particularly relevant for this work, is the presence of a well-defined hydration layer around this protein, which facilitates analysis of protein–water interactions. With a few rare exceptions, [26,54], most of the available literature data analyze the hydration of BPTI only on the one-dimensional (1D) level [55,56,57,58,59]. A detailed description of BPTI hydration on the 3D-molecular level presented in this work provides a holistic spatial insight into the hydrated state of this protein.

**Figure 1:**
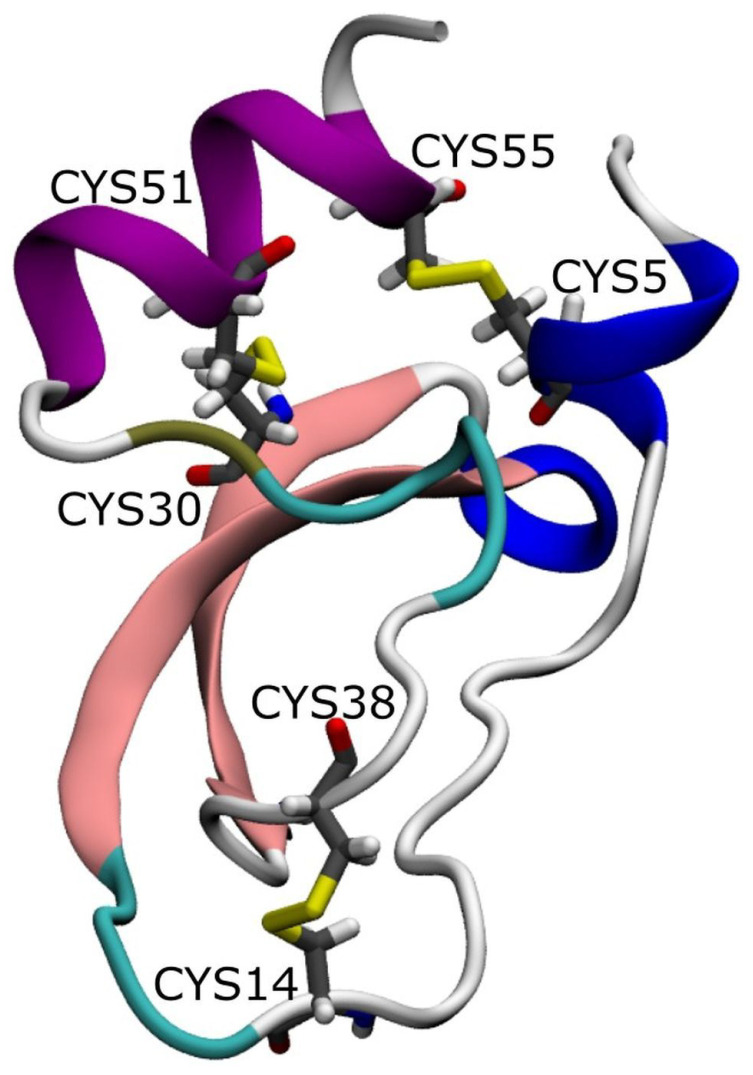
Structure of the BPTI molecule (PDB code is 5PTI) with two β-sheets, two α-helixes and two loops. The three disulfide bonds are shown by yellow.

In our study, we used the BPTI in crystal form II (PDB code is 5PTI) [35]. It contains with two β-sheets, two α-helixes and two loops (Figure 1). This protein has a net charge of +6e, which is due to the presence of the following charged residues: Arg1, Asp3, Glu7, Lys15, Arg17, Arg20, Lys26, Arg39, Lys41, Arg42, Lys46, Glu49, Asp50, Arg53, and the amino- and carboxy-termini of the polypeptide chain. As shown in [36], 59 water molecules and the four internal ones denoted usually as W111, W112, W113, and W122 are included in the X-ray and neutron structure of 5PTI. About 20 of these reside at fully occupied sites, and a few make the same H-bonds in all three crystal forms of BPTI [36]. Following Figure 1, two of the disulfide bonds (Cys5–Cys55 and Cys30–Cys51) in BPTI are completely buried in the hydrophobic core, but the third one (Cys14–Cys38) is located on the protein surface with about 50% solvent exposure of the sulfur atoms [35]. The Cys14–Cys38 disulfide bond bridges two loops, comprising residues 8–17 and 36–44 [35]. The rigidity of these loops is thought to be essential for the inhibitory function of BPTI [60,61]. In addition to three disulfide bonds, additional stabilization of the BPTI structure is provided by water molecules buried inside the protein [62] (see below Section 2.1).

## 2. Results

### 2.1. Internal Water in BPTI

Internal or, also known as buried, waters play important biological roles, including protein–ligand binding (see, for instance, the results for HIV protease [63] and hemoglobin [64]), protein–protein association [65], protein stability [66], and protein flexibility [67,68].

All three reported crystal structures of wild-type BPTI [35,36,38] contain internal water molecules, W111, W112, W113 and W122, are extensively hydrogen-bonded to main-chain atoms, forming an integral part of the native BPTI structure. The water molecule, W122, is completely buried in a very small cavity near the Cys14–Cys38 disulfide bond. Another three buried water molecules, W111, W112, and W113, form an H-bonded chain occupying a pore-like cavity with W111 at its mouth and W113 most deeply buried (approximately 7 Å away from W122) [69]. These four water molecules are also present in the solvated complex and, as indicated by several NMR relaxation studies [37,62,69,70,71], occupy the same locations as in crystal form. They have little orientational freedom and therefore move along with the protein by a factor of 2000 slower than in bulk water. [72]. The buried waters together with the other crystal waters are an indication of a somewhat ordered aqueous environment around the BPTI [73]. In fact, one can consider the internal water molecules as “internal ligands” in relation to protein.

Here, we present our MD and 3D-RISM results for internal water molecules in aqueous BPTI. It should be noted that only a few MD simulations have been able to detect such molecules [74]. Most often, MD simulation times are not long enough for their identification in a protein structure. This usually happens in too short simulations of the nanosecond order (see, for instance, [75,76,77]), and then, the buried waters are added “by hand” or not taken into account at all (see, in particular, the data for BPTI [22,55,56,62,69,74,78,79]). At the same time, NMR studies [80,81] show that the residence times of the water molecules buried in internal cavities are in the range from about 1 ns to 10 ms.

**Figure 2:**
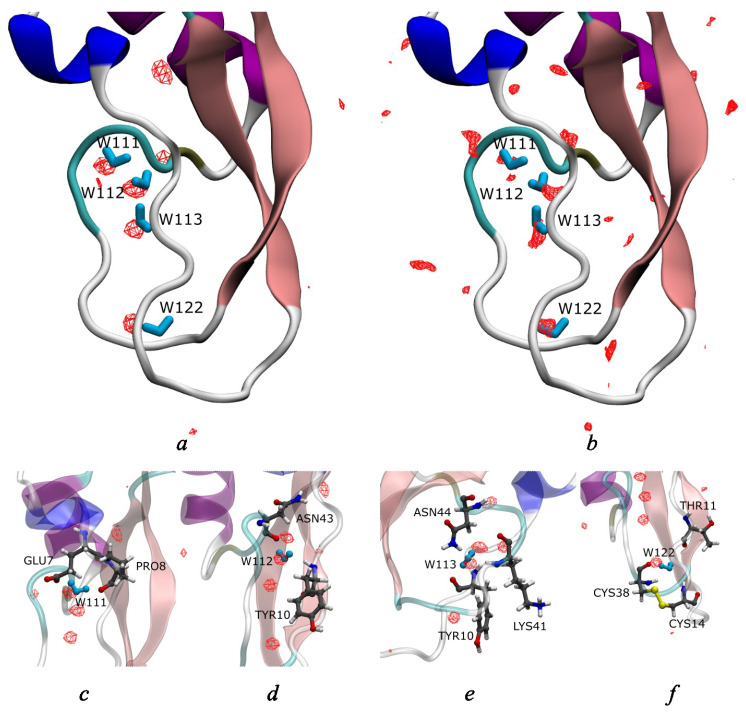
Joint location of the internal water molecules (W111, W112, W113, and W122) in structure of the BPTI molecule (PDB code is 5PTI) from (**a**) MD simulation and (**b**) 3D-RISM calculation (both in red) as well as from experiment [35] (in blue). Individual location of internal waters from MD simulation for (**c**) W111; (**d**) W112; (**e**) W113; (**f**) W122.

Four internal water molecules were found in our 2 μs MD simulation as well as from 3D-RISM calculations of aqueous BPTI (Figure 2). In both methods, the positions of rigidly bound water molecules were determined from the 3D-distribution functions of water oxygens and shown by the corresponding isosurfaces in red. As one can see from this figure, the 3D-RISM and MD results are in a good agreement with each other and with experimental data (Figure 2a,b). The buried waters are located in the positions close to those observed in the experiment [35], i.e., in the interior of the protein in the region of Glu7, Tyr10, Pro13, Arg39, and Lys41. One water molecule, W122, is a spatially isolated in a small protein cavity (Figure 2f), while three other molecules, W111, W112 and W113, form a hydrogen-bonded cluster in a large cleft (Figure 2c–e). All buried waters are hydrogen-bonded to the corresponding protein residues, namely, W122 to Cys38, Cys14, Thr11 and W111, W112 and W113 to Pro8, Tyr10, Asn43, Lys41 and Asn44 [36]. The obtained geometry of the hydrated interior of the BPTI with four internal waters and surrounding residues also coincides with results from other MD simulations [74,82].

### 2.2. BPTI Hydration

Here, we discuss the hydration structure of BPTI obtained by the 3D-RISM calculations and MD simulations focusing on analysis of the thickness of protein hydration shell. The current understanding is that only a few hundred water molecules (∼300–400) per small protein such as lysozyme or myoglobin is sufficient to form one hydration shell around the protein (see, for instance, [83,84,85,86]). Since BPTI is a small protein, we would expect its total hydration number to be in this range as well.

**Figure 3:**
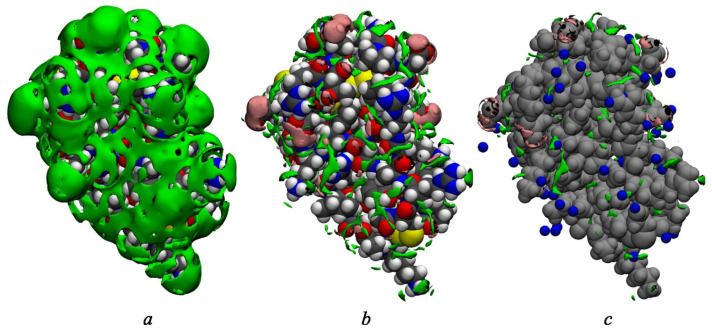
Isosurface representations of the 3D-RISM distribution function of solvent around the BPTI. (**a**) The surface of water oxygen (green) at $g_{O}\left(r\right)=2$. The protein atoms are colored in gray for C, in white for H, in red for O, in blue for N, and in yellow for S. (**b**) The surface of water oxygen (green) at $g_{O}\left(r\right)=4$ and of water hydrogen (pink) at $g_{H}\left(r\right)=3$. The protein atoms are colored in the same way as in panel (**a**). (**c**) The surface of water oxygen (green) at $g_{O}\left(r\right)=5$ and water hydrogen (pink) at $g_{H}\left(r\right)=4$ as well as water location (blue spheres) determined from the X-ray crystallography [35]. For the sake of viewability, here, all protein atoms are shown in gray.

**Figure 4:**
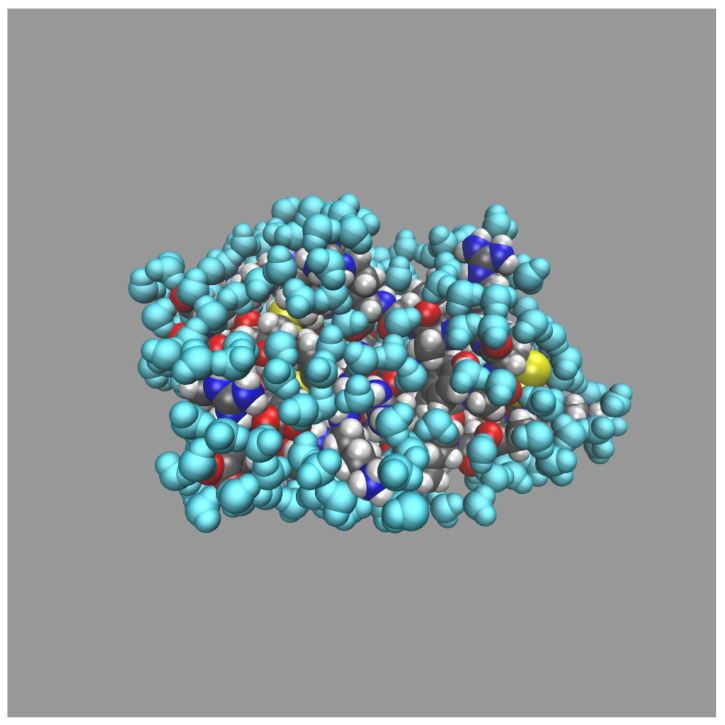
Water distribution (in cyan) around the BPTI (within 3.6 Å from the closest atoms of protein) obtained from MD simulation at the selected time of 1 μs. The colors of protein atoms are the same as in Figure 3a,b. The choice of 3.6 Å as the $r_{\mathrm{cut}}$ distance is discussed in Section 2.3.1.

The data from both methods demonstrate a well-defined hydration layer around BPTI (Figure 3a and Figure 4). The 3D-RISM approach allows us to analyze it qualitatively by considering different thresholds of isodensity surfaces (Figure 3) around the unliganded state of protein. With $g_{O}\left(r\right)=2$ (Figure 3a), we observe a broad hydration structure, which indicates the fluctuation of hydrating water. Taking into account that $g_{O}\left(r\right)=1$ in bulk, Figure 3a reflects the fact that water presence at the BPTI surface is twice as probable as in the bulk solvent. The isosurfaces of water oxygens at $g_{O}\left(r\right)=4$ and of water hydrogens at $g_{H}\left(r\right)=3$ (Figure 3b) demonstrate the localized hydration shell near the hydrophilic parts of the protein surface. The distribution of water hydrogens is found predominantly near the protein groups that can act as H-bond acceptors (carboxylate and hydroxyl groups). On the other hand, the distribution of water oxygen is found near the protein groups that can act as H-bond donors (amino and hydroxyl groups, etc.). Thus, the isosurfaces in Figure 3b can serve as evidence of H-bonding of waters from the first hydration shell with almost every exposed polar site of BPTI. A similar situation was also observed in the 3D-RISM study of lysozyme [9,87] and the single-chain variable fragment of an anti-dansyl antibody [88] in water.

Figure 3c compares the distribution of water oxygens at $g_{O}\left(r\right)=5$ obtained from 3D-RISM with water positions determined from the experiment. Ref. [35] Localized green-colored areas are indicative of water molecules strongly bound (for example, by H-bonds) to the protein (Figure 3c). Most of them correspond to water molecules found in the crystal structure of BPTI and consistent with the experimental data [35,89].

When describing the hydration structure, it is essential to define the thickness of the protein hydration shell properly, since this affects the correct determination of its hydration number. A typical prescription for its determination is to use the location of the first minimum (a cut-off distance, $r_{\mathrm{cut}}$) of the relevant radial distribution functions (RDFs). In particular, for proteins, the estimation of a shell size is often based on the distances of 4.5–5.0 Å for C atoms and 3.2–3.5 Å for O and N atoms, corresponding to the minimum of appropriate RDFs [56,90,91,92]. Another definition of the thickness of the first water layer is a single, universal cut-off distance of ∼3.5 Å for all protein (non-H) atoms [93,94,95,96,97,98] or 4.5–5.0 Å only for hydrophobic carbons of protein [26,99,100].

We apply different approaches to the definition of the width of the first hydration shell of BPTI to find the most appropriate one for this protein. We view the first hydration shell of protein as a region containing the nearest-neighbor water molecules of protein. One way to define it is to use a combination of two cut-off distances, namely, a larger distance for the non-polar radicals (4.5 Å) of hydrophobic amino acids than for the polar radicals (3.2 Å) of hydrophilic ones. Under this choice, below, we have used the location of minimum of the relevant RDFs (C_BPTI_-O_water_, N_BPTI_-O_water_, O_BPTI_-O_water_) from several MD simulations for the BPTI [54,56,57,92]. In addition, separately, we also performed calculations with the universal cut-off distance of 3.5 Å or 3.2 Å.

**Figure 5:**
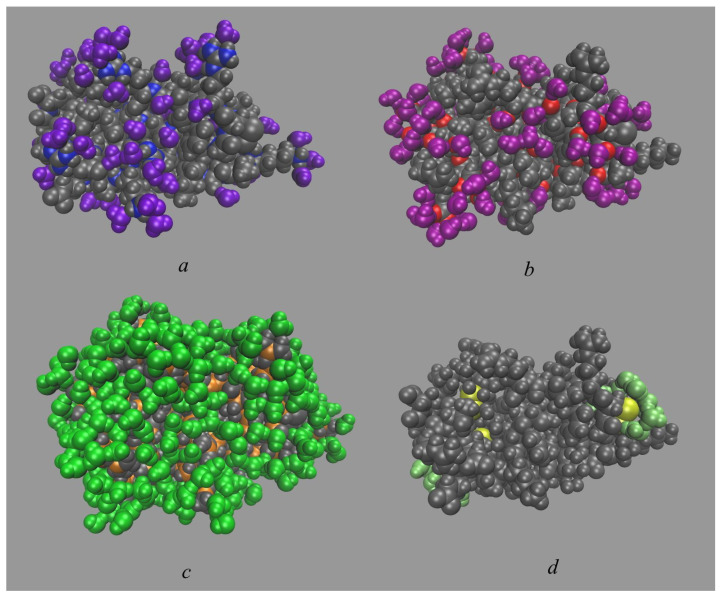
Water distribution in vicinity of the polar and non-polar regions of the BPTI at the selected time of 1 μs. The protein is colored in gray except for relevant atoms. (**a**) Hydration water molecules (in violet) within 3.2 Å from the “polar” N atoms (in blue). (**b**) Hydration water molecules (in purple) within 3.2 Å from the “polar” O atoms (in red). (**c**) Hydration water molecules (in green) within 4.5 Å from the “non-polar” C atoms (in orange). (**d**) Hydration water molecules (in lime) within 4.5 Å from S atoms (in yellow).

The simplest way to describe the BPTI hydration structure is to look at the water distribution near the polar and non-polar parts as represented by their respective atoms (N, O, and C) (Figure 5a–c). Additionally, we also consider water distribution in the vicinity of S atoms of the polar Cys (cysteine) and non-polar Met (methionine) (Figure 5d). Despite the traditional definition of Cys as a polar amino acid and Met as a non-polar amino acid, it has recently been found [101] that Cys probably has a similar hydrophobicity to Met and could serve as its equivalent replacement in protein stabilization. Therefore, here, we consider the water distribution for these sulfur-containing amino acids as a separate case at 4.5 Å like the non-polar radicals.

As can be seen from Figure 5, the use of this approach, especially, the $r_{\mathrm{cut}}$ distance of 4.5 Å for non-polar parts, results in the inclusion of not only the nearest-neighbor molecules but also water molecules from the second (and possibly higher) hydration shell. The most indicative case is water distribution in the vicinity of C atoms (Figure 5c). It also follows from the corresponding hydration numbers (Table 1), when due to the excess water molecules not from the first shell, the sum of hydration numbers for BPTI (∼554) is much larger than one would expect from estimates of the number of water molecules (∼300–400) in the first hydration layer of small proteins [83,84,85,86].

**Figure 6:**
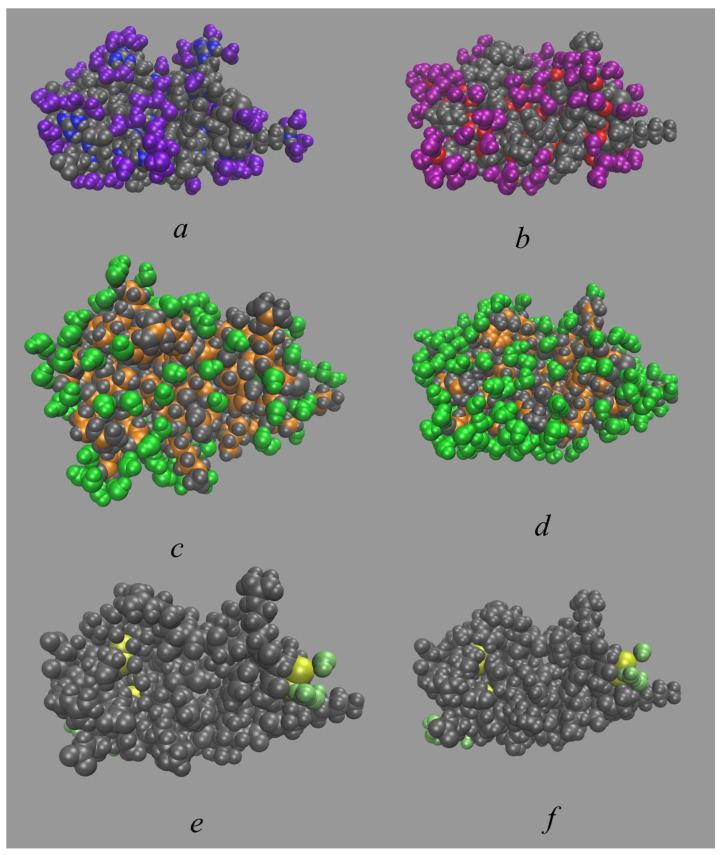
Water distribution in vicinity of the polar and non-polar regions of the BPTI at the selected time of 1 μs. The protein is colored in gray except for relevant atoms. (**a**) Hydration water molecules (in violet) within 3.5 Å from the “polar” N atoms (in blue). (**b**) Hydration water molecules (in purple) within 3.5 Å from the “polar” O atoms (in red). (**c**,**d**) Hydration water molecules (in green) within 3.2 Å (**c**) and 3.5 Å (**d**) from the “non-polar” C atoms (in orange). (**e**,**f**) Hydration water molecules (in lime) within 3.2 Å (**e**) and 3.5 Å (**f**) from S atoms (in yellow).

To “remove” the excess water molecules, we could use the distances of 3.5 Å and 3.2 Å as the universal cut-offs. These results are shown in Figure 6 and Table 2 (water distributions within 3.2 Å from the polar regions with N or O atoms are the same as in Figure 5a,b). This approach provides a more realistic picture of the BPTI hydration structure. The total hydration number of 310.4 (for 3.2 Å) and 355.3 (for 3.5 Å) (Table 2) are within the interval of the total hydration numbers estimated for small proteins (∼300–400) [83,84,85,86]. However, in the absence of general rational criterion, the choice between two universal cut-off distances for determining the shell thickness is not obvious. In the next subsection, we propose a simple and effective procedure for determining the thickness of the hydration layer of biomolecules and, as a result, for calculating their hydration numbers.

### 2.3. A Straightforward Procedure for Determining the Thickness of the Hydration Layer of Solute Molecules and Corresponding Hydration Numbers

#### 2.3.1. Application of the Procedure in the Framework of MD Method

One of the methodologies for determining the solute hydration number from the MD trajectory is the direct calculation of the number of water molecules located at a distance not exceeding a certain value, $r_{\mathrm{cut}}$. As discussed above, this value would be typically based on the location of the first minimum for the relevant RDFs, which, for BPTI, can range from 3.2 Å to 5.0 Å. As a result of such variation, there is a significant discrepancy in the estimation of hydration number.

**Figure 7:**
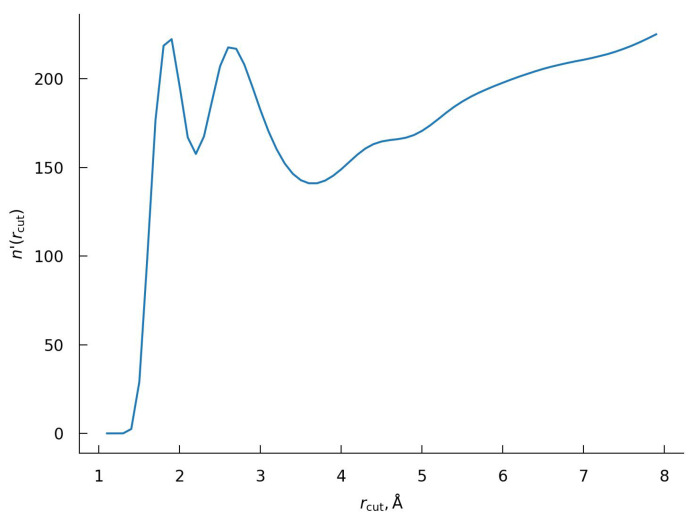
The dependence $n^{\prime }\left(r_{\mathrm{cut}}\right)$ with $r_{\mathrm{cut}}$ as the distance to the closest atoms of the BPTI defining in the framework of the MD method.

A more systematic procedure toward determination of hydration number can be developed by shifting focus toward exploring the properties of the underlying object, namely the hydration layer. Physically, the hydration layer is the region that captures the localized accumulation of the solvent molecules. As such, the hydration layer would be characterized by an increased relative density of the solvent inside the layer and a reduced one at its boundaries. The simplest way to capture this is to consider the behavior of cumulative solvent number density $n(r_{\mathrm{cut}})$—the number of solvent molecules whose nearest distance to the protein is less than a cut-off radius $r_{\mathrm{cut}}$. The rate of solvent accumulation can be then conveniently characterized by $n^{\prime }\left(r_{\mathrm{cut}}\right)=dn\left(r_{\mathrm{cut}}\right)/dr_{\mathrm{cut}}$. A similar idea has been used earlier to describe the pressure effect on the protein hydration shell [57] and to analyze the ionic distribution and affinity of various ions to protein [102]. The results obtained for the BPTI are shown in Figure 7. We note that this function is rather different from the RDF, despite some external similarity as a distribution curve.

As one can see from Figure 7, $n^{\prime }\left(r_{\mathrm{cut}}\right)$ has two pronounced peaks at distances of ∼2.0 Å and ∼2.7 Å.

**Figure 8:**
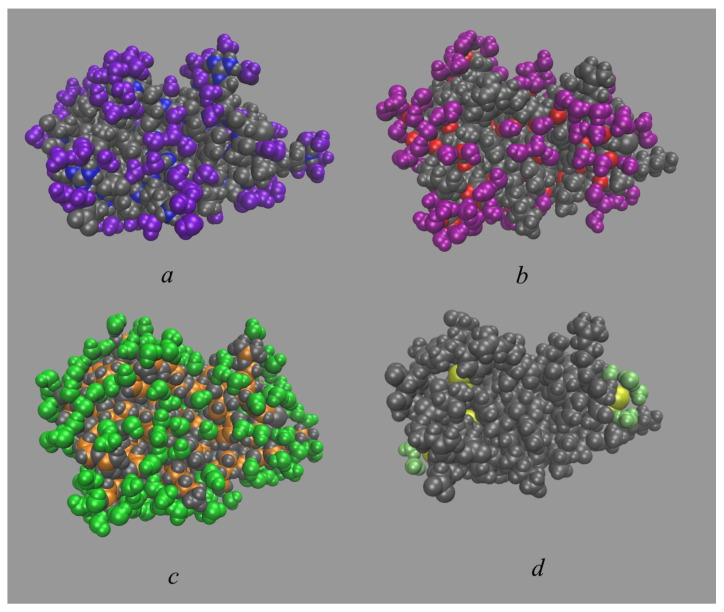
Water distribution in vicinity of the polar and non-polar regions of BPTI at the selected time of 1 μs. The protein is colored in gray except for relevant atoms. (**a**) Hydration water molecules (in violet) within 3.6 Å from the “polar” N atoms (in blue). (**b**) Hydration water molecules (in purple) within 3.6 Å from the “polar” O atoms (in red). (**c**) Hydration water molecules (in green) within 3.6 Å from the “non-polar” C atoms (in orange). (**d**) Hydration water molecules (in lime) within 3.6 Å from S atoms (in yellow).

Our interpretation is that both peaks belong to the first hydration layer, i.e., they are corresponding to water molecules near the closest atoms of protein with the first peak reflecting the H-bonding of these solvent molecules with the protein. Based on this interpretation, we accept the second minimum as the boundary of the first hydration shell with a corresponding $r_{\mathrm{cut}}$ value of 3.6 Å. Water distributions corresponding to this cut-off distance around the whole protein and in the vicinity of the polar and non-polar regions of the BPTI are shown in Figure 4 and  Figure 8 correspondingly.

Figure 4 demonstrates a pronounced hydration layer near the BPTI surface with the total hydration number of 369.4 (Table 2), which is also within the interval of the total hydration numbers estimated for small proteins [83,84,85,86]. This value is in good agreement with the value of 368.9 found in alternative MD simulation [26]. As follows from Figure 8, the solvent is more highly distributed around the polar regions than near the non-polar ones. The comparison of “individual” hydration numbers with their sum (Table 2) shows that about 55% of water molecules are in “hydration contact” with the polar parts of the protein surface. What is more, as will be shown below, most of them are located near charged polar radicals. These conclusions are confirmed by the 3D-RISM results (Figure 3), which demonstrate more preferable interactions of water molecules with polar, hydrophilic groups on the protein surface, including the H-bonding of waters with almost every exposed polar site of BPTI. This fact reflects the heterogeneity of the protein hydration shell with respect to local (“individual”) hydration numbers for the corresponding moieties. This is also supported by data on higher hydration numbers of hydrophilic groups in comparison with the hydration numbers of hydrophobic ones, which are calculated on the basis of experimental thermodynamics data [103].

**Figure 9:**
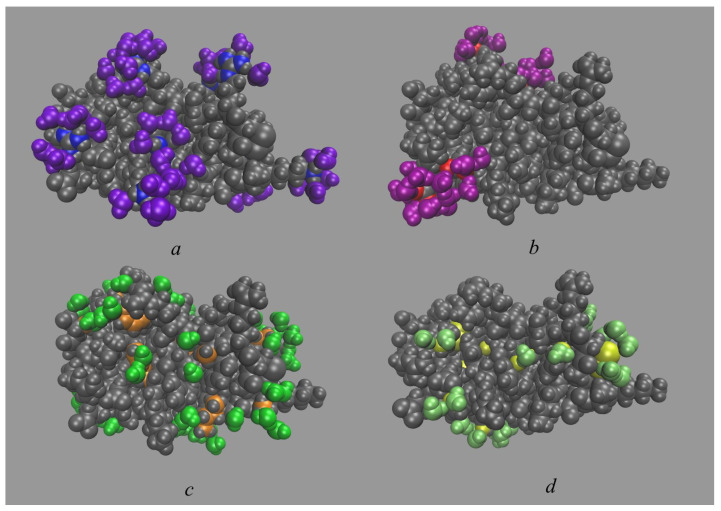
Water distribution in the vicinity of the polar charged (positively and negatively), polar uncharged, and non-polar radicals at the selected time of 1 μs. The protein is colored in gray except for relevant atoms. (**a**) Hydration water molecules (in violet) within 3.6 Å from N atoms (in blue) of amide- and amino groups of the positively charged lysine and arginine. (**b**) Hydration water molecules (in purple) within 3.6 Å from O atoms (in red) of carboxylate groups of the negatively charged aspartic and glutamic acids. (**c**) Hydration water molecules (in green) within 3.6 Å from the non-polar radicals (in orange)—from C atoms of glycine, alanine, valine, leucine, isoleucine, phenylalanine, proline and S atoms of methionine. (**d**) Hydration water molecules (in lime) within 3.6 Å from the polar uncharged radicals (in yellow)—from O atoms of hydroxyl group of serine, threonine, tyrosine, and O atoms of carbonyl group of asparagine and glutamine as well as from S atom of cysteine.

We can also give a more detailed view on hydration structure by considering the solvent distribution in the vicinity of the protein representative moieties such as the polar charged (positively and negatively), polar uncharged, and non-polar radicals. The results of such analysis for BPTI can be found in Figure 9, which shows the local solvent structure at $r_{\mathrm{cut}}$ = 3.6 Å around hydrophilic lysine and arginine with the positively charged group, hydrophilic aspartic and glutamic acids with the negatively charged group, hydrophilic uncharged serine, threonine, cysteine, asparagine, glutamine and tyrosine, and non-polar glycine, alanine, valine, leucine, isoleucine, phenylalanine, proline, methionine.

The obtained data (Figure 9 and Table 3) indicate that almost half of the water molecules are distributed near the polar charged and uncharged radicals. This fact reflects the preferable interactions of water with the polar moieties in comparison with the non-polar ones. Based on the sum of all water molecules near the polar radicals, one can determine that ∼52% of them are molecules in the vicinity of the positively charged radicals, ∼20% are molecules in the vicinity of the negatively charged radicals, and ∼28% are molecules in the vicinity of the uncharged ones (Table 3). Especially, the water molecules interact strongly with the charged moieties of the BPTI. As found [54], the energy of these interactions is −14.02 kcal/mol vs. −2.33 kcal/mol for interactions of water with the non-polar moieties. The total number of waters around the polar charged moieties is ∼100 (Table 3), which is in agreement with the other MD results [104], where it was found that sufficient hydration to cover the charged groups of the BPTI is also about 100 solvent molecules.

#### 2.3.2. Application of the Procedure in the Framework of 3D-RISM Method

Since SDFs are the main product of of 3D-RISM calculations, the total hydration number of the entire solute can be calculated by integrating the solute–water oxygen SDF, $g_{O}\left(r\right)$, as

(1) $$n=\rho_{O}\int_{V}g_{O}\left(r\right)dr,$$

where *V* is the region occupied by the first hydration shell of the entire solute molecule, and $\rho_{O}$ is the average number density of water oxygen atoms. The main difficulty for using this formula lies in determining *V*, since it depends on the shape of the solute and can have a complex form. Leaving aside initial data nuances, the trajectories in MD simulations versus the SDFs in the 3D-RISM method, the approach described above for determining the hydration shell thickness and corresponding solute hydration number can be easily applied for the case of the 3D-RISM method. To this end, we present the region of the first hydration shell, *V*, as a set of points located at a certain distance, $r_{\mathrm{cut}}$, from the solute molecule. In this case, the $r_{\mathrm{cut}}$ has the meaning of the thickness of the hydration layer. Then, the dependence of hydration number on $r_{\mathrm{cut}}$ can be also obtained and differentiated:

(2) $$n^{\prime }\left(r_{\mathrm{cut}}\right)=\frac{d}{dr_{\mathrm{cut}}}n\left(r_{\mathrm{cut}}\right)=\frac{d}{dr_{\mathrm{cut}}}\left(\rho_{O}\int_{V(r_{\mathrm{cut}})}g_{O}\left(r\right)dr\right),$$

**Figure 10:**
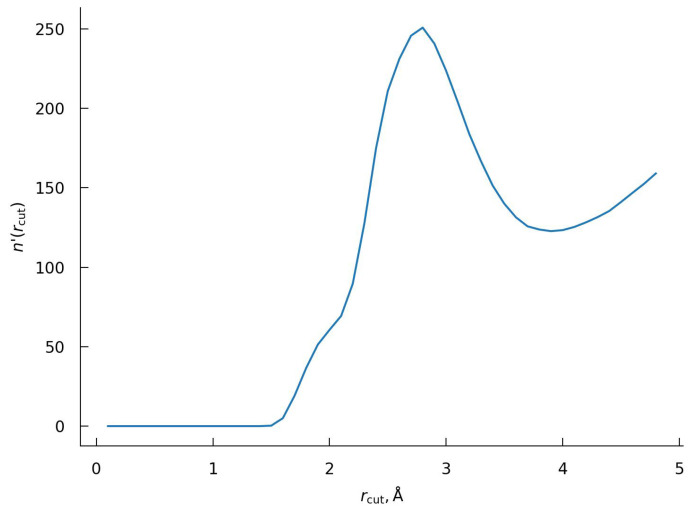
The dependence $n^{\prime }\left(r_{\mathrm{cut}}\right)$ with $r_{\mathrm{cut}}$ as the distance to the closest atoms of the BPTI. The case of 3D-RISM calculations.

Note, this procedure is proposed in the framework of the 3D-RISM method for the first time. The workability of the proposed methodology was initially tested on three amino acids such as glycine, leucine and threonine (see Table S1 and Figures S1 and S2 in Section S1 in Supplementary Materials). After confirming the effectiveness of the procedure, it was extended to the BPTI (Figure 10). As one can see from Figure 10, the function $n^{\prime }\left(r_{\mathrm{cut}}\right)$ has a wide peak at ∼2.7 Å with a shoulder at a distance of ∼2.0 Å. We accept the position of the minimum, $r_{\mathrm{cut}}$, at 3.9 Å as the boundary of the first hydration shell. The hydration number of BPTI obtained using this distance is 332.6.

## 3. Discussion

The large number of atoms and complexity of interactions makes fully atomistic analysis of hydration structure a challenging problem. Hence, in methods such as MD or classical (and site) density functional theory, the solvent structure around the proteins is usually discussed in terms of local radial distribution functions, i.e., on the 1D level. It is not clear whether such treatment can fully capture the effects related to complex protein shape. In this work, we have analyzed this issue by developing a detailed description of BPTI (PDB code is 5PTI) hydration on the 3D molecular level by utilizing a combination of 2-μs all-atom MD simulations and the 3D-RISM approach.

The structure of many proteins contains internal water molecules, which play an important role in the protein properties as noted in Section 2.1. Their detection presents a challenge for in silico methods, such as MD, due to the long simulation times required to explore these hard to reach regions in phase space. Such problems are largely absent in the 3D-RISM approach, which bypasses explicit dynamical propagation and works directly with distributions functions.

Four buried water molecules inside the BPTI were found by us in 2-μs MD simulation and from 3D-RISM calculations of aqueous BPTI. The data of both methods are in good agreement with the X-ray crystallography measurements [35] when the localization of internal waters bound firmly to the BPTI sites and their positions are corresponding to the experimental ones. Thus, the presented results indicate that the water-binding sites can predict from MD simulation and the 3D-RISM theory with great success.

Both methods have also found a well-defined hydration layer around the BPTI. While we did not study the detailed hydrogen bonding features between protein with water, the pronounced hydration layer near the protein surface assumes that a large number of water molecules are H-bonded with the BPTI by hydrogen bonds that were discussed in the literature (see, for instance, [54,57,58,105]). Wherein, the formation of hydrogen bonds will be largely determined by hydrophilic residues of protein rather than by hydrophobic ones.

At the same time, the total hydration number (the number of water molecules in the first hydration shell) of protein depends on the thickness of the first hydration layer. A typical way for the thickness definition is to take the location of the first minimum, $r_{\mathrm{cut}}$, of the relevant RDFs. A variation of the $r_{\mathrm{cut}}$ can lead to a significant discrepancy in the hydration numbers, complicating the proper comparative description of the protein hydration. As discussed in Section 2.2, depending on the chosen $r_{\mathrm{cut}}$, one may fail to include all the water molecules “in contact” with the protein surface [26] or include extraneous water molecules from the second hydration shell [90,92]. The procedure proposed in this work is based on the analysis on cumulative solvent number function $n(r_{\mathrm{cut}})$. Namely, the thickness of the hydration layer is found by looking at the point with the lowest solvent density, as conveyed by the derivative of $n(r_{\mathrm{cut}})$. Such a procedure is applicable to both MD and 3D-RISM approaches. Although the distance dependence of running hydration numbers is rather different for MD and 3D-RISM evaluations (see Figure S17 in Supplementary Materials), the obtained hydration numbers are close. In particular for MD simulation, such a procedure leads to an $r_{\mathrm{cut}}$ value of 3.6 Å with a hydration number of 369 water molecules. For 3D-RISM, we obtain 3.9 Å with ∼333 water molecules. It should be noted that despite the simplicity and efficiency of the proposed procedure, a direct comparison between the total hydration numbers obtained by two methods should be provided with caution, since not only cut-off radii are different but also the water oxygen distribution is used under the 3D-RISM calculations, whereas the water molecules entirely are being taken into account under MD simulations.

The analysis of the obtained data has also allowed us to describe some features of the BPTI hydration structure. In particular, we have found that about 55% of water molecules from the first hydration shell are in “hydration contact” with the polar parts of the protein surface. As follows from the distribution of water molecules around the polar moieties, ∼52% from them are molecules in the vicinity of the positively charged radicals, ∼20% are molecules in the vicinity of the negatively charged radicals, and ∼28% are molecules in the vicinity of the uncharged ones.

The presented results provide new insights into hydration of the BPTI in water as well as bring new knowledge to the understanding of protein hydration on the whole.

## 4. Materials and Methods

### 4.1. MD Simulation

The simulations of an aqueous solution of the BPTI were performed using the GROMACS package in version 2020.6 [106]. The initial BPTI structure was taken from the Protein Data Bank (PDB ID is 5PTI) [89]. For this structure, all the water molecules and ions were deleted, and all the deuterium atoms were replaced into hydrogens. After that, disulfide bonds between the cysteine residues were created by the LEaP program from the AmberTools 20 package [107]. The protonation states of BPTI residues were set according to the physiological pH = 7; i.e., Arg and Lys residues were protonated, whereas Asp and Glu residues were deprotonated. The force field parameters for BPTI atoms were taken from the Amber03 set [108,109]. Six chloride ions were added to BPTI to create a neutral simulation box. The obtained files with the structure and interaction parameters were converted into the GROMACS format by the ACPYPE program [110]. The final protein structure with 6523 SPC/E water molecules and six chloride ions were placed into a cubic box with a 1 nm distance between the protein surface and the box border. The chosen box size was enough to perform the correct simulations of solvated BPTI.

The energy of the solvated BPTI was minimized using the steepest descent algorithm. After that, the system was equilibrated in an NVT ensemble at 300 K with the V-rescale thermostat (modified Berendsen thermostat) for 0.1 ns. At the next stage, the system was equilibrated in the NPT ensemble at 300 K and 1 bar with a V-rescale thermostat and a Parrinello–Rahman barostat for 0.1 ns. After the equilibration, a 2 μs production run in the NPT ensemble was performed at 300 K and 1 bar with the same thermostat and barostat. The time step in all the equilibration and production simulations was 0.2 fs.

**Figure 11:**
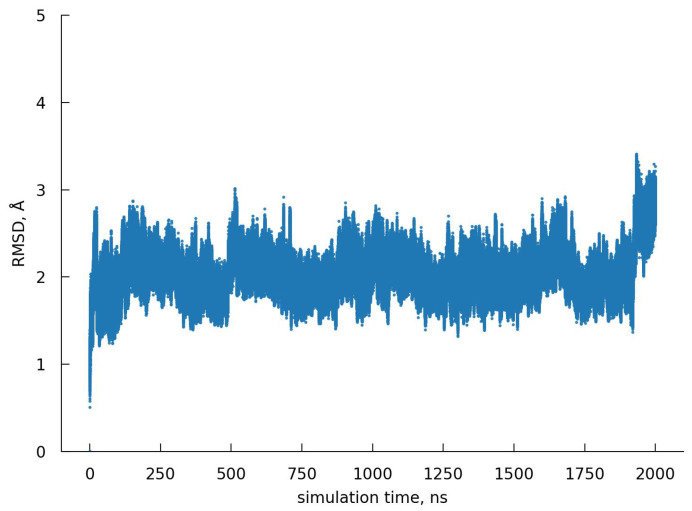
RMS displacement of the BPTI atoms from the initial structure during the main simulation.

As the protein hydration structure depends on its conformation, it is crucial to understand conformational changes of the protein before studying its hydration shell. We evaluated the conformational changes by calculating the RMS displacement of the current structure from the initial geometry (Figure 11). Figure 11 clearly shows that the protein structure remains almost unchanged during the simulation, which means any time point can be used to study the instantaneous structure of the BPTI hydration shell. Additionally, to indicate that the protein is well equilibrated and the BPTI hydration shell does not change at different time points, the overlay of BPTI structures at 0.5, 1.0, 1.5, and 2.0 μs as well as the dependence of the number of water molecules around the protein on the time of MD simulation are presented in Figures S3–S15 in the Supplementary Materials. According to the above, we have chosen the 1 μs time point as the middle of the main simulation, and thus, the MD snapshots presented in the text are according this time.

Under determination of the total hydration number, we have calculated the number of water molecules located at a distance less than or equal to $r_{\mathrm{cut}}$ from at least one of the BPTI atoms. These values were evaluated every picosecond throughout the entire simulation. The average of the obtained set of values was taken as the total hydration number of the protein. A similar procedure was used to calculate the “individual” hydration numbers, i.e., the hydration numbers for relevant regions of protein (Table 1, Table 2 and Table 3). However, in this case, it was taken into account that the distances between water molecules and BPTI atoms are corresponding to a certain criterion (for example, to a certain type of atoms, belonging to the polar or non-polar type of amino acid residues, etc.). Thus, the total hydration number is not the sum of “individual” hydration numbers (Table 2 and Table 3).

### 4.2. Three-Dimensional (3D)-RISM Calculations

As the background of the 3D-RISM theory was already described elsewhere [111,112,113,114], we give here only briefly some details of this approach as well as of computational details relevant for our study. The 3D-RISM method provides a description of the solute hydration structure on the molecular-atom level based on the solute–solvent interactions. The 3D-RISM theory operates with the molecule–atom spatial distribution functions (SDFs), $g_{\alpha }\left(r\right)$, for solvent sites/atoms α around the reference entire solute molecule. The SDF $g_{\alpha }\left(r\right)\equiv g_{\alpha }\left(r,\Omega \right)$ is the three-dimensional (3D) density distribution function of solvent atoms in a local coordinate system linked with the solute molecule. To obtain the SDF, one fixes a solute molecule at the origin of a local (spherical) reference frame and characterizes the local atomic densities by computing both the radial *r* and angular Ω=(θ,φ) coordinates of the vector r. The SDF is often presented in terms of isodensity surfaces at some probability level, for instance, relative to bulk system. These functions are the result of the numerical solution of the 3D-RISM Ornstein–Zernike (OZ) equation with the appropriate closure relation. Here, we have used 3D-RISM OZ [114] coupled with the 3D-Kovalenko–Hirata closure [115] to obtain the SDFs. The used Kovalenko–Hirata (KH) closure is a coupling of the mean spherical approximation for the regions of density enrichment ($g_{\alpha }\left(r\right)>1$) with the hypernetted chain approximation for the region of density depletion ($g_{\alpha }\left(r\right)<1$). This closure is among the best closure relations to date in terms of both numerical stability and reasonable accuracy [116,117]. As pushed in [117], a substantial advantage of the 3D-KH closure approximation is that it properly accounts for the solvation structure of complex solvated systems with significant association effects. At the same time, the 3D-hypernetted chain (HNC) closure strongly overestimates such associative effects, and therefore, the 3D-RISM-HNC equations diverge in many practical applications for macromolecules with considerable site charges solvated in polar solvents or electrolyte solutions. Other closures such as the Percus–Yevick, modified Verlet, Martynov–Sarkisov, and Ballone–Pastore–Galli–Gazzillo closures do not properly account for the electrostatic asymptotics of the interaction potential [117].

BPTI hydration structures in water were calculated at ambient conditions using the rism3d.snglpnt code from the AmberTools 20 package [107]. The numerical solution of the 3D-integral equations was performed by the MDIIS (Modified Direct Inversion in the Iterative Subspace) iterative scheme [118]. A rate of convergence for the solution of RISM equations was controlled by the number of MDIIS vectors (10) and the value for a mixing parameter (the MDIIS step size) of the solution. The 3D-RISM equations were solved on a three-dimensional grid of 320 × 270 × 294 points and with a spacing of 0.025 nm in a cell of size (8.00 × 6.75 × 7.35) nm^3^. A residual tolerance of 10^−6^ was chosen that was set to be enough to obtain the structural data with accuracy of 10${}^{-3}$. The grid size chosen is large enough to accommodate the protein together with sufficient solvation space around them so that the obtained results are without significant numerical errors.

The stable structure of BPTI in water (Figure S16 in Supplementary Materials) used in 3D-RISM calculations was obtained from 2 μs MD simulation described above. The partial charges and the corresponding Van der Waals parameters for protein atoms were taken from the Amber ff14SB parameter set [119]. For water, the modified version of the SPC/E model (MSPC/E) was used [120].

## Figures and Tables

**Table 1:** Hydration numbers with standard deviations for the polar and non-polar regions with the relevant protein atoms (N, O, C and S). Cut-off distances are 3.2 Å and 4.5 Å for the polar and non-polar parts, respectively.

Regions with Relevant Protein Atoms	Hydration Number
Polar regions with N atoms	80.9±5.9
Polar regions with O atoms	97.4±5.8
Non-polar regions with C atoms	357.2±10.0
Regions with S atoms	18.4±3.2

**Table 2:** Hydration numbers with standard deviations for the polar and non-polar regions with the relevant protein atoms (N, O, C and S). Universal cut-off distances are 3.2 Å and 3.5 Å as well as 3.6 Å for both polar and non-polar parts. The $r_{\mathrm{cut}}$ of 3.6 Å was obtained as described in Section 2.3.1.

Regions with Relevant Protein Atoms	Hydration Number (3.2 Å)	Hydration Number (3.5 Å)	Hydration Number (3.6 Å)
Polar regions with N atoms	80.9±5.9	120.0±7.1	132.8±7.5
Polar regions with O atoms	97.4±5.8	118.2±7.0	127.4±7.5
Non-polar regions with C atoms	106.1±6.8	177.9±9.0	203.3±9.3
Regions with S atoms	3.1±1.5	5.9±2.0	6.9±2.2
**Total hydration number**	310.4±10.4	355.3±10.7	369.4±10.8

**Table 3:** Hydration numbers with standard deviations for regions with the relevant protein radicals. Universal cut-off distance is 3.6 Å as described in Section 2.3.1.

Regions with Relevant Protein Radical	Hydration Number (3.6 Å)
Polar positively charged radicals	72.3±5.5
Polar negatively charged radicals	28.5±3.8
Non-polar radicals	76.8±7.4
Polar uncharged radicals	39.3±4.1
**Total hydration number**	369.4±10.8

## Data Availability

Not applicable.

## Supplementary Materials

The following supporting information can be downloaded online https://www.mdpi.com/article/10.3390/ijms232314785/s1.
