# Supplementary material for: Protein 3D Hydration: A Case of Bovine Pancreatic Trypsin Inhibitor
Source: Int J Mol Sci. 2022 Nov 26;23(23):14785. doi: 10.3390/ijms232314785 (PMC9737982; doi:10.3390/ijms232314785)
Supplement: Supplementary file 1 [file ijms-23-14785-s001.zip › ijms-2024356-supplementary.pdf]

Supplementary Materials: Protein 3D-Hydration: a Case of Bovine Pancreatic Trypsyn Inhibitor

Sergey E. Kruchinin [1], Ekaterina E. Kislinskaya [2], Gennady N. Chuev [3,*] and Marina V. Fedotova [1,*]

1. Test of workability for the procedure of determining the thickness of the hydration layer of solute molecules and corresponding hydration numbers in the framework of 3D-RISM method

Test systems are amino acids glycine (Gly), leucine (L-Leu) and threonine (L-Thr) in ZW form (Figure S1).

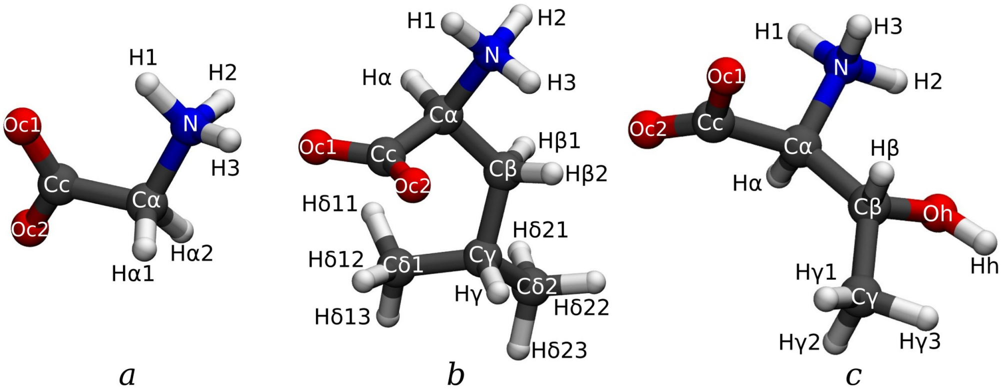

Figure S1. Spatial structure of Gly, L-Leu, and L-Thr with atom numbering.

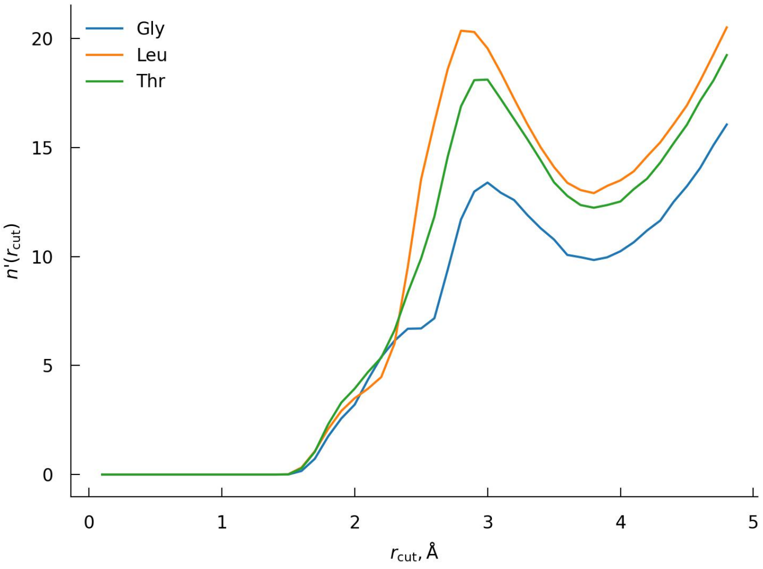

Figure S2. The dependence $n'(r_{cut})$ as the distance to the closest atoms of Gly, L-Leu, and L-Thr calculated by the proposed procedure in the framework of 3D-RISM method (See the text in Main Manuscript).

The thickness of the hydration layer of Gly, L-Leu, and L-Thr was defined as the location of the first minimum on the corresponding functions $n'(r_{cut})$ (Figure S2). Then this distance was used to calculate the total hydration numbers of amino acids under study that were compared with those from MD simulations (Table S1).

Table S1. Hydration numbers of Gly, L-Leu, and L-Thr calculated for the r_{cut} values determined from the first minimum of the $n'(r_{\mathrm{cut}})$ (first column), for the general value r_{cut} of 3.8 Å (second column) and according to the MD simulation (third column).

**Solute**	$n_{\mathrm{3D-RISM}}(r_{\mathrm{cut}})$	$n_{\mathrm{3D-RISM}}(r_{\mathrm{cut}} = 3.8)$	$n_{\mathrm{MD}}(r_{\mathrm{cut}})$
Glycine	18.6 (3.9)	17.7	14.5
Leucine	25.6 (3.8)	25.6	22.3
Threonine	22.1 (3.7)	23.3	19.1

As can be seen from the data in Table S1, the r_{cut} values for various amino acids practically do not differ and are in the range of 3.7-3.9 Å (first column, the values in the parentheses). Therefore, we have also used a general value r_{cut} of 3.8 Å to calculate the hydration numbers. As one can see from Table S1, the hydration numbers of Gly, L-Leu, and L-Thr calculated with the individual r_{cut} values and general value r_{cut} of 3.8 Å are close. They are also in good agreement with the corresponding values hydration numbers obtained from MD simulations. A discrepancy in the 3D-RISM and MD values is due to the fact that the water oxygen distribution is used under the 3D-RISM calculations whereas the water molecules are taking into account entirely under MD simulations.

2. A comparison of the structures of BPTI and its hydration surroundings at several time points under MD simulation

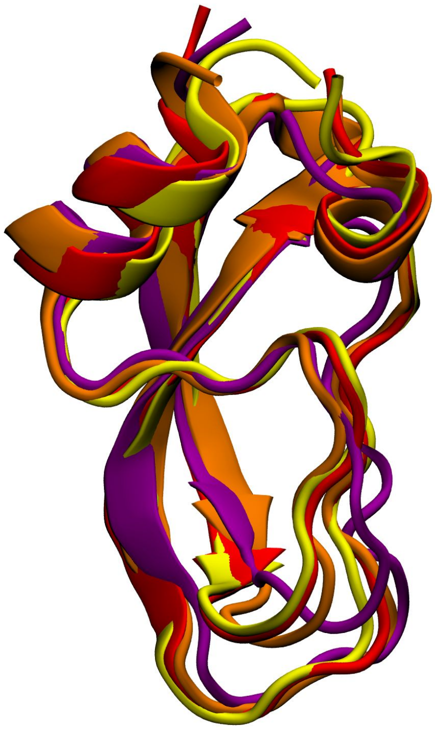

Figure S3. MD simulation: Superposition of the BPTI protein structure at 500 ns (yellow), 1000 ns (orange), 1500 ns (red), 2000 ns (magenta).

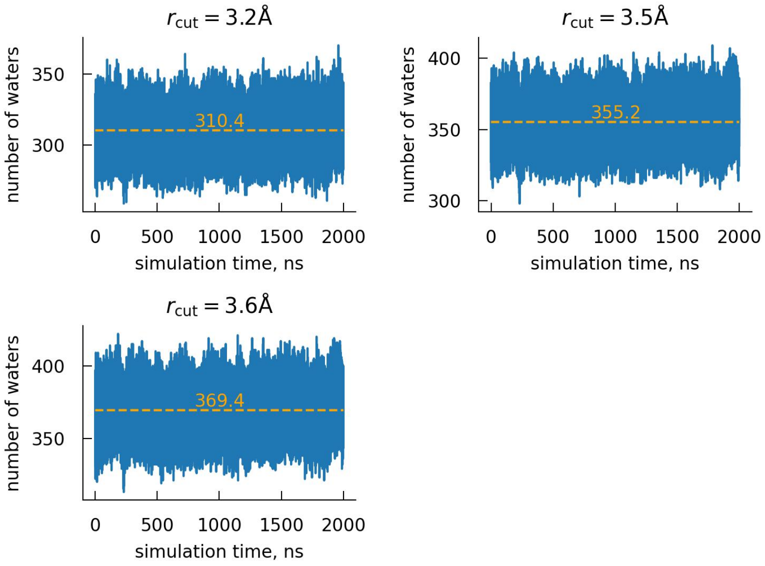

Figure S4. Dependence of the number of water molecules around the protein on the time of MD simulation at various r_{cut}. Orange color shows the average values corresponding to the total hydration numbers at various r_{cut} (values in the row "Total hydration number" in Table **??**).

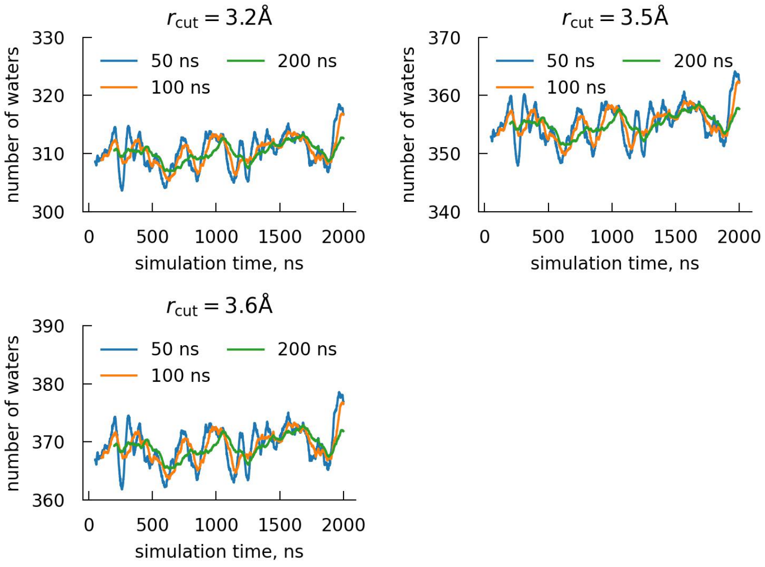

Figure S5. Running averages with different window widths (50, 100 and 200 ns) of the number of water molecules around the protein on the time of MD simulation at various r_{cut}.

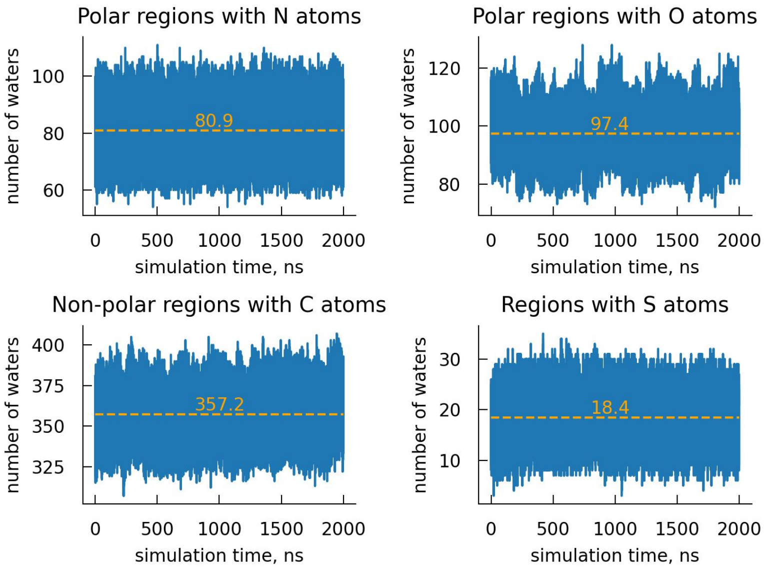

Figure S6. Dependence of the number of water molecules for the polar and non-polar regions with the relevant protein atoms (N, O, C and S) on the time of MD simulation at $r_{\text{cut}} = 4.5$ Å (polar parts) and $r_{\text{cut}} = 3.2$ Å (non-polar parts). Orange color shows the average values corresponding to the partial hydration numbers (values in Table **??**).

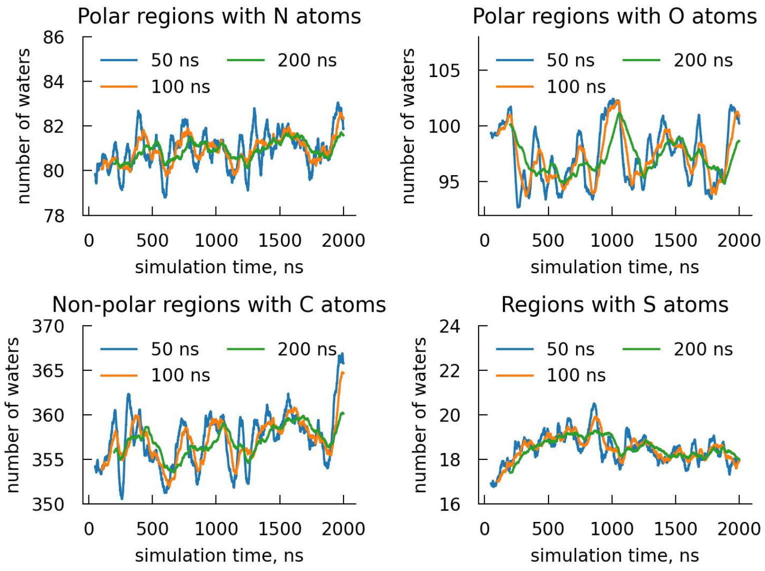

Figure S7. Running averages with different window widths (50, 100 and 200 ns) of the number of water molecules for the polar and non-polar regions with the relevant protein atoms (N, O, C and S) on the time of MD simulation at $r_{\text{cut}} = 4.5$ Å (polar parts) and $r_{\text{cut}} = 3.2$ Å (non-polar parts).

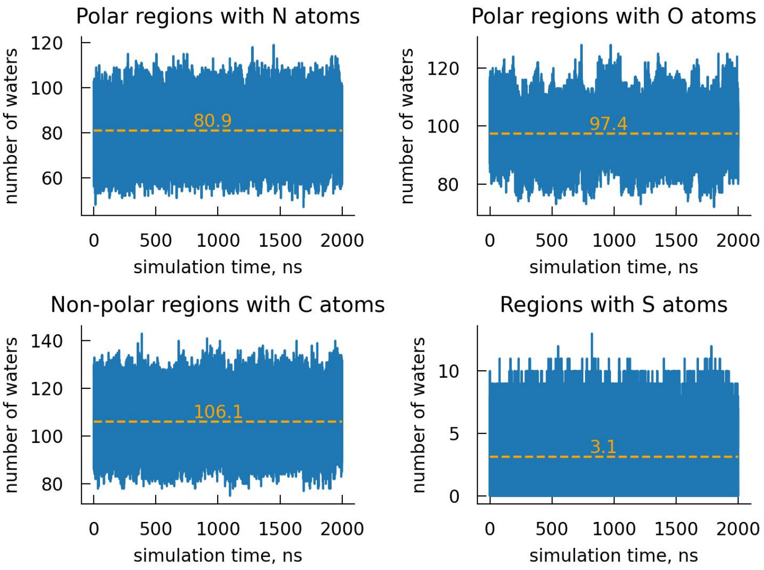

Figure S8. Dependence of the number of water molecules for the polar and non-polar regions with the relevant protein atoms (N, O, C and S) on the time of MD simulation at $r_{\text{cut}} = 3.2$ Å. Orange color shows the average values corresponding to the partial hydration numbers (values in the first column in Table **??**).

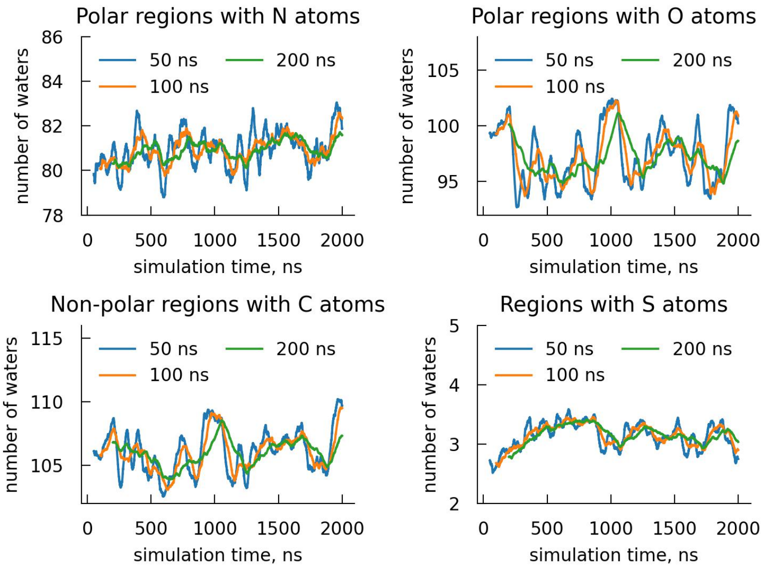

Figure S9. Running averages with different window widths (50, 100 and 200 ns) of the number of water molecules for the polar and non-polar regions with the relevant protein atoms (N, O, C and S) on the time of MD simulation at $r_{\text{cut}} = 3.2$ Å.

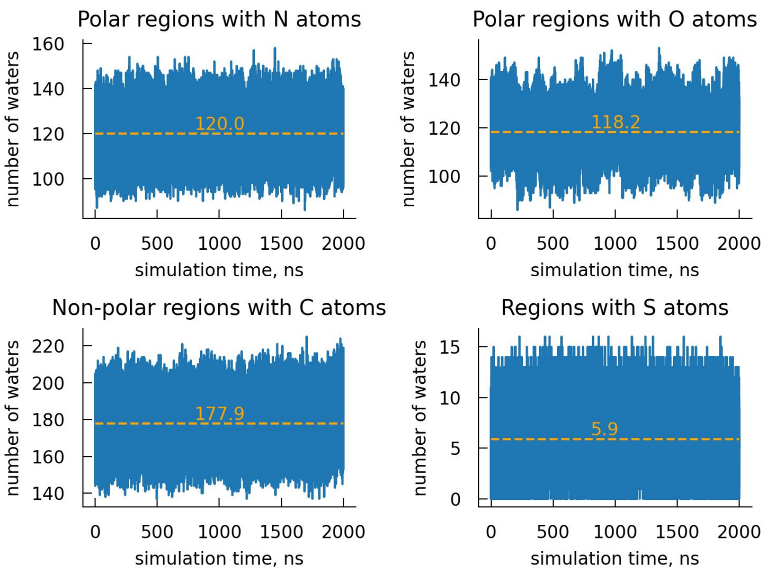

Figure S10. Dependence of the number of water molecules for the polar and non-polar regions with the relevant protein atoms (N, O, C and S) on the time of MD simulation at $r_{\text{cut}} = 3.5$ Å. Orange color shows the average values corresponding to the partial hydration numbers (values in the second column in Table **??**).

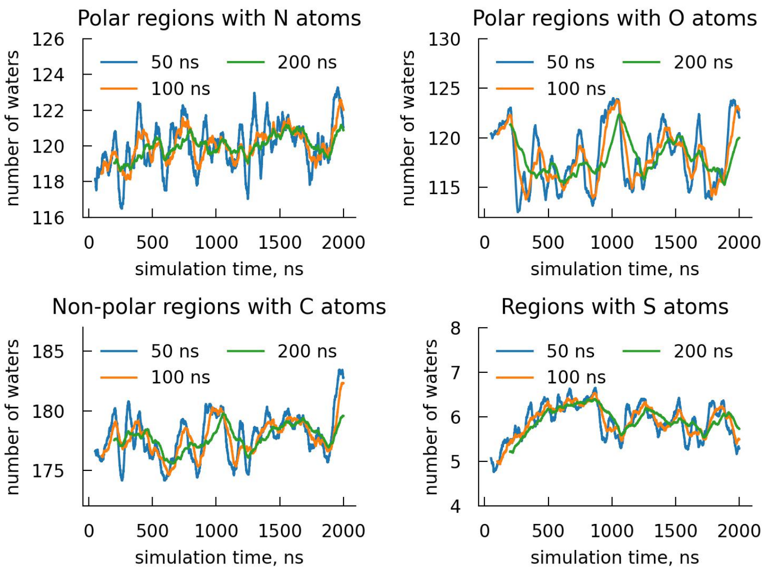

Figure S11. Running averages with different window widths (50, 100 and 200 ns) of the number of water molecules for the polar and non-polar regions with the relevant protein atoms (N, O, C and S) on the time of MD simulation at $r_{\text{cut}} = 3.5$ Å.

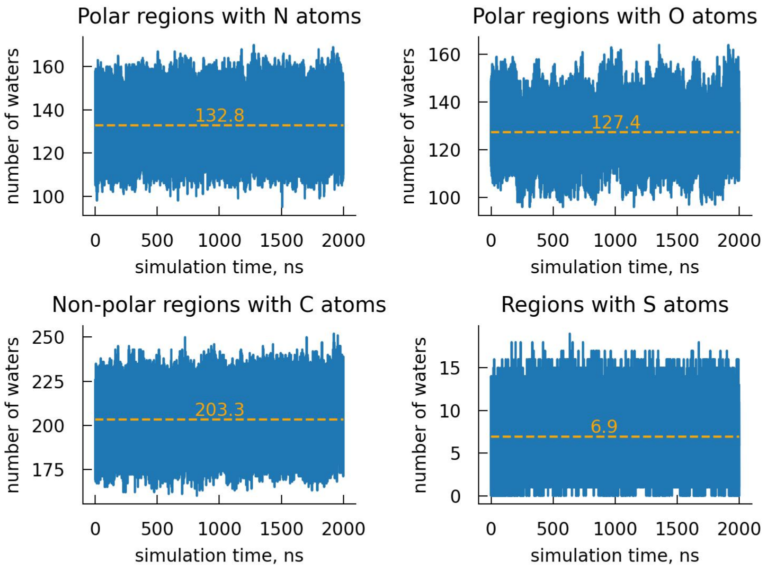

Figure S12. Dependence of the number of water molecules for the polar and non-polar regions with the relevant protein atoms (N, O, C and S) on the time of MD simulation at $r_{\rm cut} = 3.6$ Å. Orange color shows the average values corresponding to the partial hydration numbers (values in the third column in Table **??**).

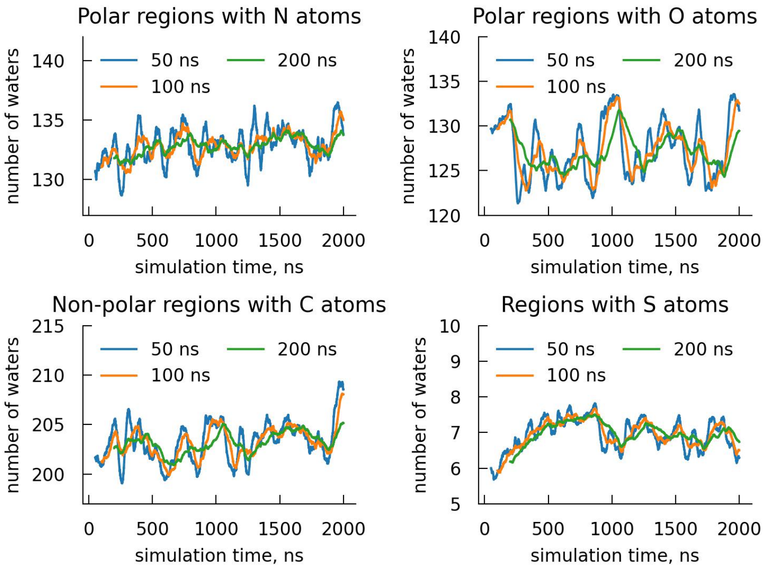

Figure S13. Running averages with different window widths (50, 100 and 200 ns) of the number of water molecules for the polar and non-polar regions with the relevant protein atoms (N, O, C and S) on the time of MD simulation at $r_{\rm cut} = 3.6$ Å.

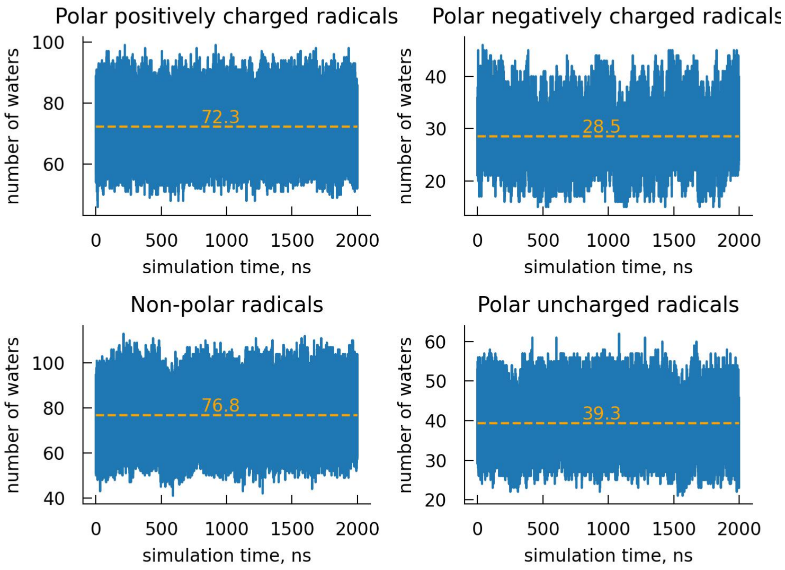

Figure S14. Dependence of the number of water molecules for for regions with the relevant protein radicals on the time of MD simulation at $r_{\text{cut}} = 3.6$ Å. Orange color shows the average values corresponding to the partial hydration numbers (values in Table **??**).

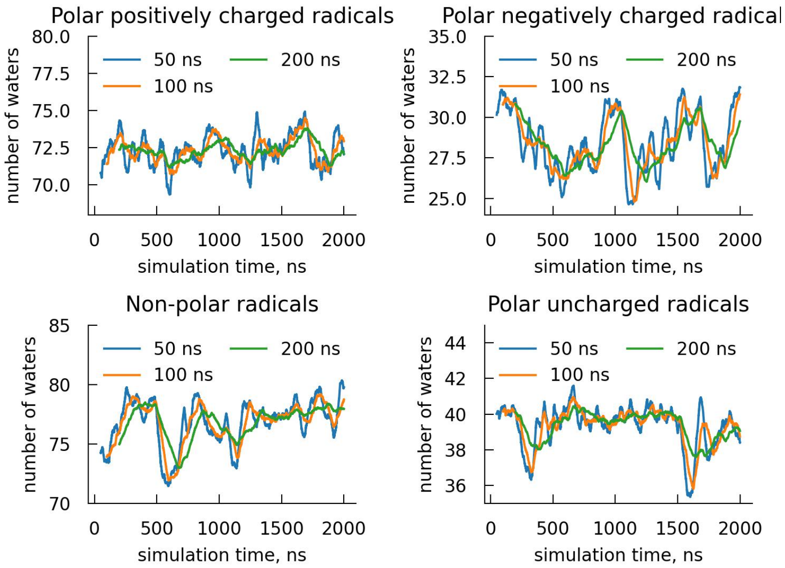

Figure S15. Running averages with different window widths (50, 100 and 200 ns) of the number of water molecules for for regions with the relevant protein radicals on the time of MD simulation at $r_{\text{cut}} = 3.6$ Å.

3. Spatial configuration of BPTI used in 3D-RISM calculations 21

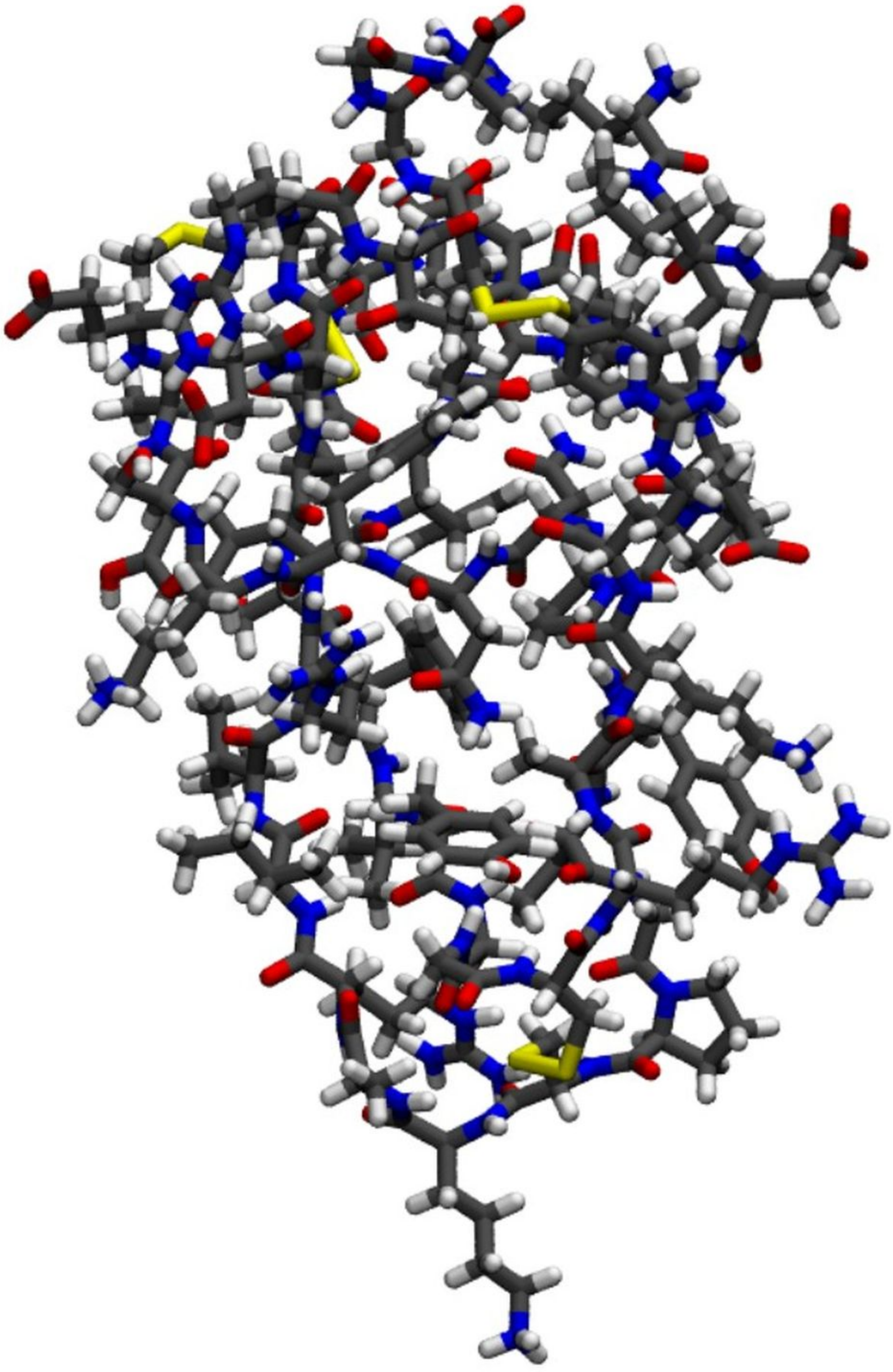

Figure S16. Spatial configuration of BPTI used in 3D-RISM calculations. The protein atoms are colored in gray for C, in white for H, in red for O, in blue for N, in yellow for S.

4. Dependence of $n(r_{cut})$

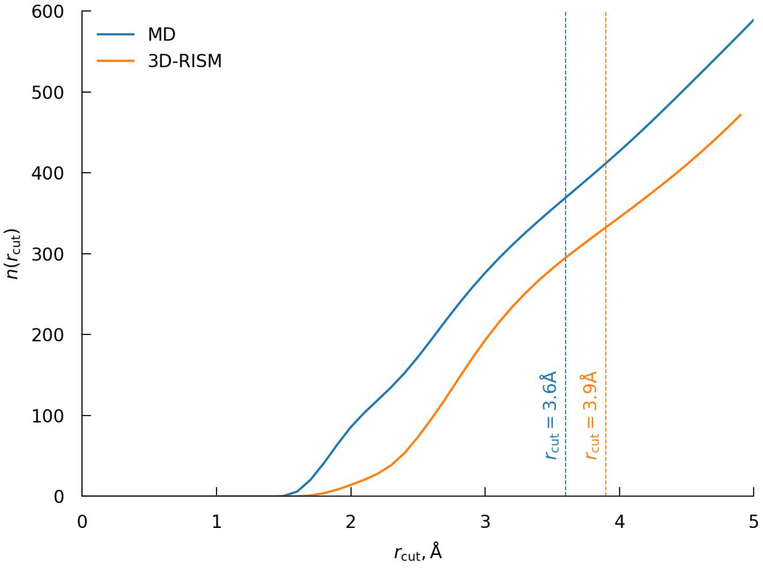

Figure S17. Dependence of $n(r_{cut})$ obtained from MD (blue) and 3D-RISM (orange) data. The r_{cut} values used in the paper are shown as vertical dashed lines.
